# IL1 Pathway in HPV-Negative HNSCC Cells Is an Indicator of Radioresistance After Photon and Carbon Ion Irradiation Without Functional Involvement

**DOI:** 10.3389/fonc.2022.878675

**Published:** 2022-04-22

**Authors:** Dinesh Kumar Tiwari, Ricarda Hannen, Kristian Unger, Sibylla Kohl, Julia Heß, Kirsten Lauber, Florentine S. B. Subtil, Ekkehard Dikomey, Rita Engenhart-Cabillic, Ulrike Schötz

**Affiliations:** ^1^ Department of Radiotherapy and Radiooncology, Philipps-University Marburg, Marburg, Germany; ^2^ Research Unit Radiation Cytogenetics, Helmholtz Center Munich, German Research Center for Environmental Health GmbH, Neuherberg, Germany; ^3^ Department of Radiation Oncology, University Hospital, Ludwig-Maximilians-University (LMU) München, Munich, Germany; ^4^ Clinical Cooperation Group “Personalized Radiotherapy in Head and Neck Cancer”, Helmholtz Zentrum München, Neuherberg, Germany

**Keywords:** HNSCC, radioresistance, senescence, SASP, IL1, photon, carbon ion, tumor

## Abstract

**Background:**

Treatment of locally advanced HPV-negative head and neck squamous cell carcinoma (HNSCC) with photon radiation is the standard of care but shows only moderate success. Alterations in response toward DNA DSB repair, apoptosis, and senescence are underlying determinants of radioresistance in the tumor cells. Recently, senescence and the associated secretory phenotype (SASP) came into the focus of research and raised the need to identify the tumor-promoting molecular mechanisms of the SASP. The aim of this project was to unravel more of this process and to understand the impact of the IL1 pathway, which plays a major role in SASP. The studies were performed for photon and ^12^C-ion irradiation, which strongly vary in their effect on radioresistance.

**Materials and Methods:**

A panel of five HPV-negative HNSCC cell lines was treated with photon and ^12^C-ion irradiation and examined for clonogenic survival, DNA DSB repair, and senescence. SASP and IL1 gene expressions were determined by RNA sequencing and activation of the IL1 pathway by ELISA. A functional impact of IL1A and IL1B was examined by specific siRNA knockdown.

**Results:**

Cell killing and residual DSBs were higher after ^12^C-ion than after photon irradiation. ^12^C-ion induced more senescence with a significant correlation with cell survival. The impact on radioresistance appears to be less than after photon irradiation. The expression of SASP-related genes and the IL1 pathway are strongly induced by both types of irradiation and correlate with radioresistance and senescence, especially IL1A and IL1B which exhibit excellent associations. Surprisingly, knockdown of IL1A and IL1B revealed that the IL1 pathway is functionally not involved in radioresistance, DSB repair, or induction of senescence.

**Conclusions:**

IL1A and IL1B are excellent indicators of cellular radioresistance and senescence in HNSCC cells without functional involvement in these processes. Clearly more research is needed to understand the molecular mechanisms of senescence and SASP and its impact on radioresistance.

## Introduction

Ionizing radiation with photons is a main pillar in the treatment of locally advanced head and neck squamous cell carcinoma (HNSCC). The disease poses a severe threat to the patient, and treatment is challenging. Locoregionally advanced stages in the HPV-negative setting experience multimodal treatment strategies composed of surgery, radiotherapy (RT), and concurrent chemotherapy, albeit with modest outcome (1). Locoregional relapse is the most prominent failure with up to 40%, limiting 8-year overall survival to approximately 50% in HPV-negative cases (2). A resistance toward irradiation of these tumors is made responsible for therapeutic failure and raises the need for optimization of RT (3). The standard-of-care treatment cannot be further intensified, since it is associated with severe side effects in the anatomically complex area of the head and neck, reducing quality of life for many patients (4). Instead, alternative strategies are investigated, to improve survival and reduce toxicity.

Radiation exposure causes genomic stress and induces a wide variety of lesions, with DNA double-strand breaks (DSBs) representing the most toxic lesion. An inevitable consequence of unrepaired DNA DSBs that are passed through the cell cycle is an aberrant mitosis culminating in mitotic catastrophe. This intermediate cellular state is a highly common event after irradiation of solid tumors, and it will be resolved to a certain degree by intrinsic apoptosis, but more importantly by cellular senescence (5, 6). The irradiation-induced senescence and the accompanied NFκB-dependent secretion of inflammatory factors of the senescence-associated secretory phenotype (SASP) are of specific interest, since accumulating evidence by us and other authors suggests their involvement in therapy resistance (7–9).

Senescence is well described to be a feature of cellular stress response toward many tumor therapies, including irradiation, and can occur in both tumor and normal cells. In recent years, it has been more and more understood that the metabolic reprogramming taking place in senescent cells can contribute to therapy resistance in a cell autonomous or non-cell autonomous manner by propagation of tumor cell survival, proliferation, and stemness (10–13). SASP secretion is regulated on the transcriptional level by a variety of transcription factors, but NFκB makes a pivotal contribution to the response (14, 15). The transcribed SASP mRNA is stabilized by MAPKAPK2 (16), and mTOR regulates the SASP on the translational level by controlling IL1A mRNA translation (17). Within SASP, IL1A and IL1B hold a key position by autocrine activation of the IL1 pathway, which stimulates the NFκB-mediated transcription of SASP genes (18–21).

In a recent study, we demonstrated the capability of modulating the cytotoxic powers of IR on HNSCC cells by interfering with the SASP *in vitro* and *in vivo* (9). In the present study, we investigated the role of the SASP factors IL1A and IL1B for tumor cell killing by IR in the context of irradiation-induced senescence *in vitro*. Besides photons, we used particle irradiation with carbon (^12^C) ions. The technology of particle beam delivery enables an enhanced relative biological effectiveness (RBE) in the Bragg peak leading to improved inactivation of tumor tissue in a highly precise defined area, and the physical properties of the beam reduce unwanted effects to normal tissue (22). Ongoing clinical trials demonstrate the advantages of ^12^C-ion irradiation and encourage preclinical research into this area to open this treatment option to a wider community (23–26). The tumor-reducing capabilities of ionizing irradiation are interlinked with the DNA damage response, cell death mechanisms, and cellular stress responses to activate the removal of damaged cells, while responsiveness of these pathways toward irradiation increases with increasing LET (27, 28). Since ionizing irradiation of varying LET was described to result in various levels of senescence (29), we selected these two different radiation qualities to study the molecular mechanisms of senescence and SASP in the context of IL1 on different levels.

A panel of five HNSCC cell lines was irradiated with photons and ^12^C-ions and examined for clonogenic survival, DNA damage, senescence, and SASP induction as well as IL1 pathway activation. As expected, irradiation with ^12^C-ions resulted in a higher RBE and reduced DNA damage repair than observed after photons. Senescence induction was stronger after ^12^C-ions and exhibited a high and significant association with clonogenic survival. Expression of SASP factors and IL1 pathway activation were strongly correlated with clonogenic survival and senescence, yet more pronounced after ^12^C-ions. Surprisingly, functional studies of IL1A and IL1B using specific siRNA knockdown revealed that these two factors are not involved in the mechanisms controlling radioresistance. Overall, the study demonstrates that the IL1 pathway, although being a strong indicator of radioresistance and senescence in HNSCC cells after both photon and ^12^C-ion irradiation, is functionally not involved in these processes.

## Methods

### Cell Culture

The five HPV-negative cell lines Cal27, Cal33, UPCI:SCC040, UPCI:SCC131, UPCI:SCC099 (ATCC, Wesel and DSMZ, Braunschweig, Germany) were cultured in DMEM (Life Technologies, Darmstadt, Germany) supplemented with 10% fetal bovine serum (Capricorn, Ebsdorfergrund, Germany) and maintained at 37°C in a humidified 5% CO_2_ atmosphere. The GenePrint 10 kit (Promega, Walldorf, Germany) and GeneMapper 5.0 software were used for short-tandem repeat analysis. To confirm the authenticity of all cell lines, the data were compared to Expasy and DSMZ databases (30). Cell lines are routinely tested free from mycoplasma contamination using a PCR-based assay (31).

### Carbon Ion Irradiation

Cell lines and samples were irradiated at the Marburg Ion-Beam Therapy Centre (MIT) with a vertical beam of 114.5–129.5 MeV/n ^12^C-ions and positioned in a spread-out Bragg peak (SOBP) of 10–20 mm as described previously (32). Fields were applied using active scanning with a field size of 18 × 18 cm^2^. Doses of 0.25, 0.5, 1, 2, 3, and 4 Gy were applied as indicated.

### Photon Irradiation

An X-ray biological irradiator Precision X-RAD 320ix (Precision X-Ray, North Branford, CT, USA) was used to irradiate cell lines and samples at 320 kV and 8 mA, dose rate of 1.1 Gy/min, and Thoräus filter 0.5 mm Cu+ 0.5 mm Al. Absolute dose measurements confirmed the applied doses. Doses of 1, 2, 4, 6, and 8 Gy were applied as indicated.

### Colony Formation Assay

For the preplating assay (33), exponentially growing single cells were seeded in a range of 1 × 10^1^–10^4^ cells/cm^2^, depending on the cell line and irradiation dose. Cells were irradiated after adherence with the indicated irradiation quality and dose.

For the delayed plating assay, exponentially growing cells were seeded in a range of 5 × 10^4^—10^5^ cells/cm^2^, depending on the cell line. Cells were irradiated after adherence with the indicated irradiation quality and dose. 16 hours after irradiation, growing single cells were seeded in a range of 1 × 10^1^–10^4^ cells/cm^2^.

After incubation for 10–14 days, the grown colonies were fixed and stained (10% formaldehyde, 0.1% crystal violet) and colonies >50 cells were counted. The clonogenic survival was calculated by normalization to the plating efficiency of untreated cells. Survival curves were fitted to the linear–quadratic model (SF = exp - [a × D + b × D^2]) according to a least square fit (GraphPad Prism 8.1.1 software, San Diego, CA, USA). D10 was extrapolated from these data and used for calculation of RBE. Each experiment was done at least in three biological triplicates with a minimum of three independent technical repetitions.

### Senescence

Senescence was measured by detection of senescence-associated β-galactosidase (SA-βgal) activity with a flow cytometer (34). Cells were seeded at 2–4 × 10^4^ cells/cm^2^, irradiated after adherence at the indicated doses, and incubated for 1–6 days. Staining was done with 5-dodecanoylaminofluorescein-di-β-galactopyranoside (C12-FDG, Thermo Scientific, Waltham, MA, USA) after lysosomal alkalinization with bafilomycin A1, which ensures the lysosomal origin of SA-βgal activity. The experiment was described previously (9). In deviation from this study, all cells with high C12-FDG and high SSC signal were considered senescent and the setup of flow cytometric parameters in terms of photodiode settings varied, explaining the difference in total intensities of senescence, but showing similar amounts of relative senescence. All measurements were performed on a LSRII cytometer (BD Biosciences, Heidelberg, Germany), and data were analyzed with FlowJo v10 Software (Tree Star Inc., Ashland, OR, USA). Each experiment was done in biological triplicates.

### DSB Repair Foci

DSB repair foci were analyzed using co-staining of γH2AX and 53BP1, as described previously (32). Cells were fixed and stained at 0, 4, and 24 h after irradiation with a primary antibody solution as follows: mouse monoclonal anti-phospho-S139-H2AX antibody (1:500, clone JBW301, Millipore, Darmstadt, Germany) and rabbit polyclonal 53BP1 antibody (1:500, Novus Biologicals, Wiesbaden, Germany). Secondary antibody solutions were mouse Alexa-Fluor 594 (1:1,000) and rabbit Alexa-Fluor 488 (1:1,000, both Invitrogen, Karlsruhe, Germany). Cells were mounted in ProLong Gold Antifade Reagent with DAPI (Invitrogen, Karlsruhe, Germany). Immunofluorescence was analyzed using the Leica DM5500 wide-field microscope and LAS-AF software (Leica, Wetzlar, Germany). All experiments were performed at least twice and with 100 counted nuclei per experiment.

### Elisa IL1B

Briefly, 1–1.5 × 10^5^ cells/10 cm^2^ were grown in 2 ml medium and irradiated after a 16 h adherence with photons or ^12^C-ions as indicated. Supernatants were collected on indicated days after irradiation, immediately frozen in liquid nitrogen, and kept at -80°C until further analysis. Cytokine concentrations in cell culture supernatants were measured with the human IL1B DuoSet ELISA System (R&D Systems, Minneapolis, MN, USA) according to the manufacturer’s protocol. All experiments were performed twice in technical duplicates.

### Downregulation of IL1A/IL1B by RNA Interference

Transient knockdown of the transcripts was performed using Lipofectamine 2000 (Life Technologies, Carlsbad, CA) as described previously (35). Cells were seeded in a density of 1.5 × 10^4^ cells/cm^2^ and transfected after a 16 h adherence with 50 nM siRNA, according to the manufacturer’s protocol. Human IL1A or IL1B and control siRNA oligonucleotides (ON-TARGETplus, SMARTpool) were purchased from Dharmacon (Horizon Discovery Group, Cambridge, UK). Sequences are listed in **Supplementary Table 1**. Knockdown efficiency was verified by qRT-PCR 1 day after transfection.

### qRT-PCR and RNA Sequencing

Cells were seeded at 5 × 10^4^ cells/cm^2^ and treated and RNA extracted as indicated (dose and time point) using NucleoSpin RNA II Kit (Macherey-Nagel, Dueren, Germany) according to the manufacturer’s instructions. cDNA was reverse transcribed by incubation of 500 ng RNA with 200 U RevertAid Reverse Transcriptase in the presence of 5 μM random hexamers, 5 μM Oligo(dT)_18_, 500 μM dNTPs, and 1 U/μl RiboLock RNase inhibitor (all from Thermo Scientific). qRT-PCR was carried out on a QuantStudio5 Real-Time PCR System (Thermo Scientific, Germany). Relative quantification was calculated by the ddCT method. For normalization, a housekeeper reference gene was used. Primer sequences are stated in **Supplementary Table 2**.

Transcriptome generation and data preprocessing were conducted as described in Hirschberger et al. (36). In brief, sequencing libraries were prepared using the QuantSeq 3′ mRNA-Seq Library Prep Kit FWD for Illumina (Lexogen GmbH, Vienna, Austria), while the optimal number of amplification cycles was determined using the PCR Add-on kit for Illumina (Lexogen GmbH, Austria). Prior to sequencing on an Illumina HiSeq 4000 machine, the quantity and quality of libraries were assessed using the Quanti‐iT PicoGreen dsDNA Assay Kit (Invitrogen, Carlsbad, CA, USA) and the Bioanalyzer High Sensitivity DNA Analysis Kit (Agilent Technologies, Inc., Santa Clara, CA, USA). An equimolar pool of libraries was prepared and sequenced in 150 bp paired-end mode. Preprocessing of data included adapter trimming, followed by alignment to the human genome (GRCh38) and counting of reads per gene. Only genes with a total read count greater than five times the sample size were kept in the data set. Generation of variance-stabilized expression values was performed using the DESeq2 R Bioconductor package (37).

### Statistical Analysis

Graphs: Data were visualized using mean values and standard deviations over all individual experiments. GraphPad Prism version 8.1 (GraphPad Software, San Diego, CA, USA) was used for statistical analysis. Student’s t-test statistics were applied with significant p-values < 0.05.

Area under curve (AUC): for correlation analysis, AUC was determined for each individual cell line on linear survival curves for the clonogenic survival data sets, and on linear time kinetics for the senescence data sets. The total area and SD values are given in **Supplementary Tables 3, 4**.

RNA seq: Qlucore Omics Explorer v3.7 (Qlucore, Lund, Sweden) was used to analyze RNA sequencing data based on vst expressions. Principal component analysis (PCA) was performed on z-scaled data. Hierarchical clustering was used to visualize genes differentially expressed between irradiated and unirradiated samples (absolute log2-fold change > 1.5, Benjamini–Hochberg false discovery rate (FDR) < 0.05).

## Results

### Impact of Photon and ^12^C-Ion Irradiation on Cell Killing

**Figure 1:**
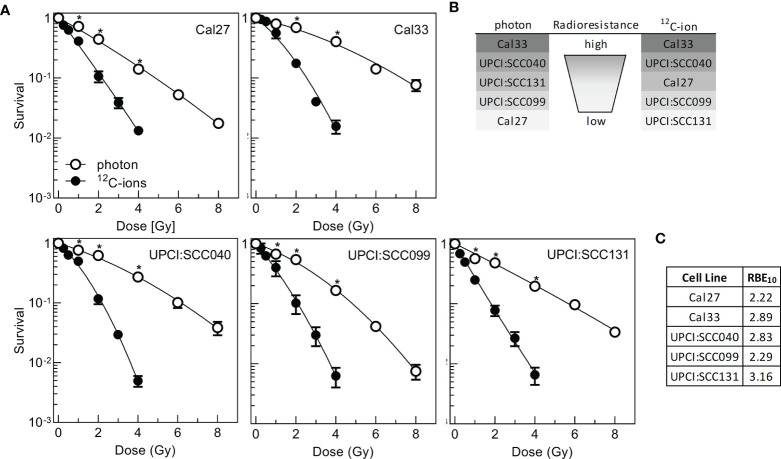
Radioresistance of five HPV-negative HNSCC cell lines after photon- or ^12^C-ion irradiation. Exponentially growing cells were irradiated and incubated for 16 h followed by delayed plating for colony formation. **(A)** Clonogenic survival after irradiation. **(B)** Ranking of radioresistance according to the AUC calculated from linear presentation of dose–response curves. **(C)** Relative biological effectiveness at 10% survival (RBE_10_). Experiments were performed at least three times. Mean +/- SEM are indicated. Significance levels were calculated with Student’s t-test statistics and significant p-values below 0.05 marked with an asterisk.

To understand the relevance of senescence and SASP in HNSCC cells, radioresistance was determined after two types of radiation with different efficacies in cytotoxic cell killing. Cells were exposed to 2, 4, 6, or 8 Gy photon or 0.25, 0.5, 1, 2, 3, or 4 Gy ^12^C-ion irradiation, and survival was scored *via* colony formation assay (**Figure 1A**). For the five cell lines used, there was a considerable variation in radioresistance, which was more pronounced after photon irradiation (**Supplementary Figure 1A**). To express the specific radioresistance by applying the entire dose–response curve, area under curve (AUC) values were calculated for the linear presentation of the data (**Supplementary Figure 1B**, **Supplementary Table 3**). Cal33 and UPCI:SCC040 are the two most radioresistant cell lines after both radiation qualities, while Cal27 is the most sensitive cell line after photon but of intermediate radioresistance after ^12^C-ion irradiation, and the opposite is seen for UPCI:SCC131; UPCI:SCC099 is the second sensitive cell line for both photons and ^12^C-ions (**Figure 1B**).

Data were also used to calculate the relative biological effect at 10% survival (RBE10). The values obtained varied from 2.22 to 3.16 showing that on average ^12^C-ions are two to three times more effective than photons (**Figure 1C**), which is well in line with previous data (32).

The results obtained for photons are in good congruence with a previous study (9), separating a radioresistant (Cal33, UPCI:SCC040) from a rather radiosensitive cluster (other cell lines). Nonetheless, some differences in the detailed ranking are seen, which might be due to the fact that delayed plating was used instead of preplating and cellular cooperation is a strong determinant of assay performance (38, 39) but also that after photon irradiation the overall variation in radioresistance was rather small. To overcome this deficit, now also ^12^C-ions were used, enhancing the overall variation in radiation response.

### Diminished Double-Strand Break Repair Efficiency After ^12^C-Ion Irradiation

**Figure 2:**
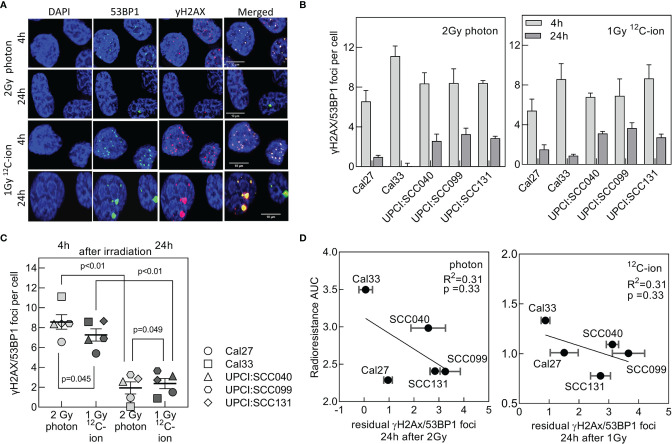
DSB repair efficiency after photon and ^12^C-ion irradiation. Cells were exposed to 2 Gy photons or 1 Gy ^12^C-ions and after repair incubation for 4 and 24 h DSBs were detected *via* γH2AX/53BP1 co-localization. **(A)** Representative pictures for immunofluorescence co-staining of foci with 53BP1 (green), γH2AX (red), and counterstaining of the nucleus with DAPI (blue). Cell line: Cal33. **(B)** Number of co-localizing foci 4 and 24 h after irradiation after background subtraction (foci at 0 Gy). **(C)** Comparison of repair efficiencies between photon and ^12^C-ion irradiation. Values are presented as MV +/- SEM after background subtraction (foci measured in unirradiated samples). p values were calculated using t-test statistics. p < 0.05 are considered significant. **(D)** Correlation of radioresistance AUC with residual γH2AX/53BP1 foci for photon and ^12^C-ion irradiation.

DNA-DSBs are the most critical damage induced by irradiation when left un- or misrepaired. Therefore, DSB repair is considered to be a main terminator of radioresistance. DSB repair was studied after the isoeffective doses of 2 Gy photon and 1 Gy ^12^C-ion. DSBs were detected 4 and 24 h after irradiation *via* γH2AX/53BP1 foci colocalization (**Figure 2A**). For both radiation qualities, all cell lines exhibited an efficient repair as indicated by the significant reduction in foci with increasing repair incubation (**Figure 2B** and **Supplementary Figure 1C**). On average, the number of initial foci measured 4 h after irradiation was slightly lower for ^12^C-ion irradiation (**Figure 2C**), which is due to the lower physical dose. However, despite this difference in dose significantly more residual foci are found 24 h after ^12^C-ion irradiation, indicating a clearly less efficient DSB repair when compared to photons. This lower DSB repair efficiency is considered to contribute to the higher effect of ^12^C-ions on cell survival. For both radiation qualities, there was a moderate correlation of residual foci with radioresistance AUC (**Figure 2D**). The correlation did not reach significance but is in good agreement with previous data from our lab (32). In conclusion, residual foci are an indicator of radiosensitivity but are not sufficient to explain the variations in radiation response of the HNSCC cell-line panel.

### Stronger Induction of Senescence After ^12^C-Ion Irradiation

**Figure 3:**
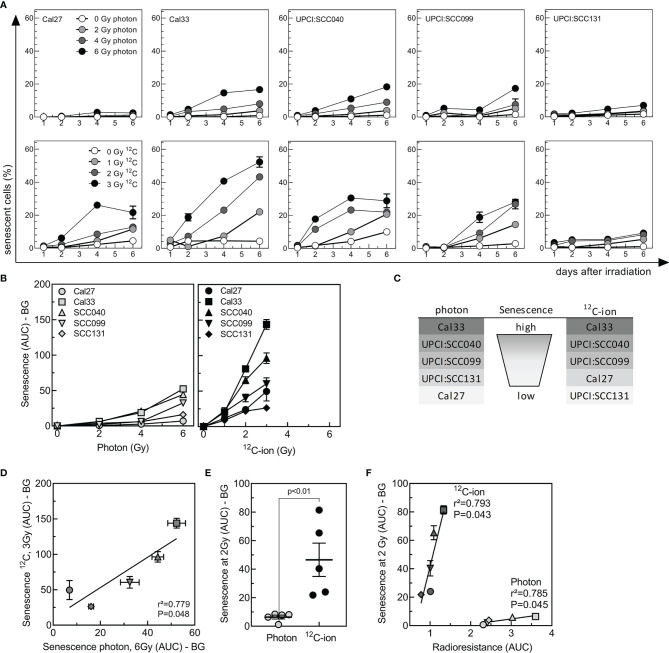
Senescence induced by photon or ^12^C-ion irradiation in HNSCC cell-lines. Cells were irradiated either with 2-, 4, or 6 Gy photons or 1-, 2-, or 3-Gy ^12^C-ions, and senescence was detected for 6 days by flow-cytometric measurement of SA-βgal activity. **(A)** Kinetics of senescence as a function of dose and time after irradiation. **(B)** Senescence (AUC with background BG subtracted) as a function of dose. **(C)** Ranking of senescence induced by irradiation. **(D)** Correlation between senescence induced by 6-Gy photons or 3-Gy ^12^C-ions. **(E)** Group analysis of senescence (AUC-BG) induced by 2-Gy photons or ^12^C-ions. **(F)** Association between senescence induced by 2 Gy of photons or ^12^C ions (AUC-BG) with the radioresistance (AUC) of the respective cell line. Values are indicated as MV ± SEM. p values were calculated using t-test statistics. p values < 0.05 are considered significant.

Cellular senescence is a frequent event after irradiation, to prevent propagation of damaged cells. Due to metabolic changes, senescent cells switch their expression profiles toward inflammatory factors summarized as SASP, which might also induce radioresistance (40). To evaluate senescence, cells were irradiated with 2–6 Gy photon or 1–3 Gy ^12^C-ions and senescence-associated lysosomal β-galactosidase activity (34) was determined by flow cytometry over a period of 6 days after irradiation (**Supplementary Figure 2**). The fraction of senescent cells increased from day 2 after irradiation over time and with dose but showed large variations between cell lines and radiation qualities (**Figure 3A**).

Senescence was quantified by calculating AUC (**Figure 3B**). Cal33 and UPCI:SCC040 showed a strong induction after both photon and ^12^C-ion, while UPCI:SCC099 presented intermediate senescence levels and UPCI:SCC131 and Cal27 were characterized by very low senescence (**Figures 3B, C** **)**. Overall, there was a strong correlation for the senescence induced by these two types of irradiation (**Figure 3D**), with a much stronger effect after ^12^C-ion irradiation. For an identical dose of 2 Gy, senescence was about 7 times higher after ^12^C-ions when compared to photons (**Figure 3E**). The correlation of senescence AUC and radioresistance AUC revealed a strong and significant association of the two parameters for ^12^C-ions and similar results for photons (**Figure 3F**). It is noticeable that the slope of the association was much steeper for ^12^C-ion irradiation. Albeit the strong increase in senescence, radiosensitivity did not increase to the same extent. This suggests that overall senescence appeared to have a lower impact on radioresistance after ^12^C-ions than it did after photons.

### For Photon and ^12^C-Ion Irradiation Activation of SASP Genes and IL1 Pathway Correlate With Radioresistance

**Figure 4:**
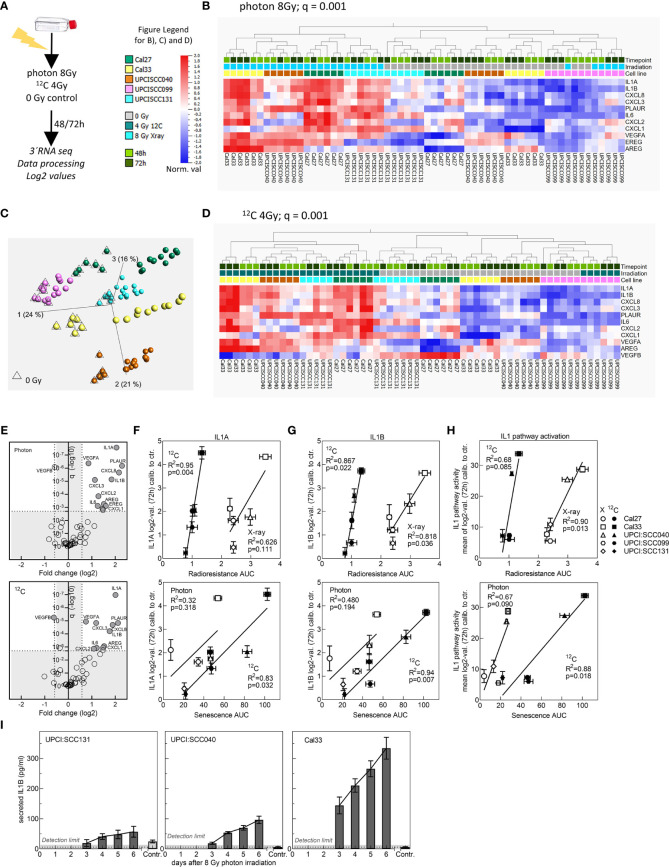
Evaluation of SASP gene expression after irradiation with 8 Gy photons or 4 Gy ^12^C-ions. **(A)** Schematic of experimental procedure. **(B)** Principal component analysis (PCA) on z-scaled data of the five cell lines for the 49 SASP factors. **(C)** Hierarchical clustering (fold change >1.5) for 11 genes to separate irradiated from unirradiated samples for photons and **(D)** ^12^C-ions. **(E)** Plot depicting fold change and q value for significantly differently expressed genes between unirradiated and irradiated samples for photons and ^12^C-ions. **(F)** IL1A and **(G)** IL1B values 72 h after irradiation were calibrated to the unirradiated control and correlated with radioresistance AUC or senescence AUC. **(H)** Correlation of IL1 pathway activity with radioresistance AUC or senescence AUC. Experiments were performed in three biol. replicates. Values are MV+/- SEM. T-test statistics were used for p value calculation. p < 0.05 are considered significant. R^2^: Pearson coefficient. For PCA and hierarchical clustering, log2-transformed, normalized (mean = 0, var = 1) data was used. Benjamini–Hochberg correction was applied, and q < 0.05 was considered significant. **(I)** Detection of IL1B protein secretion by ELISA up to 6 days after irradiation with 8 Gy photons in the cell lines UPCI:SCC131, UPCI:SCC040, and Cal33.

Senescent cells are characterized by an altered expression profile summarized as SASP. Members of the SASP include chemokines and cytokines, but also growth factors, which are secreted and enable communication with the microenvironment (41). We used RNA sequencing data to examine the gene expression profiles of SASP family members in the cell line panel. The cell lines were irradiated with 8 Gy photons or 4 Gy ^12^C-ions or mock irradiated, and RNA was extracted 48 and 72 h after irradiation (**Figure 4A**). 3′ sequencing of the RNA (RNA-seq) was performed, data were preprocessed, and log2 values were extracted. After QA, from the RNA-seq dataset, 49 SASP genes could be extracted for further analysis (**Supplementary Table 4**). PCA on z-scaled data revealed a cell-line-specific SASP gene expression profile (**Figure 4B**). It can also be observed that unirradiated samples with low SASP can be distinguished from irradiated samples with high SASP. Separation is not completely achieved for UPCI:SCC131 and UPCI:SCC099 cells. UPCI:SCC131 unirradiated cells express low but noticeable SASP levels and cannot be clearly distinguished from irradiated samples, while for UPCI:SCC099 SASP is not induced after irradiation and, as a consequence, all samples appear in the unirradiated cluster (**Figures 4B–D**). 8 Gy photons and 4 Gy ^12^C-ions induce highly similar expression patterns for almost all factors, and hence samples cannot be separated according to radiation quality. Using hierarchical clustering, irradiated samples can be separated from unirradiated samples in case of photons (q = 0.001; **Figure 4C**) as well as ^12^C-ions (q = 0.001; **Figure 4D**) by an 11-gene identifier. These genes are expressed significantly differently between irradiated and unirradiated samples with a fold change >1.5 (**Figure 4E**) and include members of the IL1 pathway, various chemokines, and growth factors. Correlation studies for IL1A (**Figure 4F**) and IL1B (**Figure 4G**) show strong associations for these genes with radioresistance and senescence, where the associations are always stronger for ^12^C-ions. This analysis demonstrates that IL1A and IL1B gene expression are strong indicators for the radioresistance of HPV-negative HNSCC cells. The result was confirmed using qRT-PCR analysis of independent samples (**Supplementary Figure 3A–C**).

Since both proteins are known to activate the identical pathway, we were interested to examine other relevant genes of the IL1 pathway. 25 genes could be extracted from the RNA-seq data set (**Supplementary Table 5**), and similar to the analysis with the SASP genes, hierarchical clustering was able to separate irradiated from unirradiated samples for photons (q = 0.006; **Supplementary Figure 4A**) and ^12^C-ions (q = 0.001; **Supplementary Figure 4B**). Separation was excellent for Cal27, Cal33, and UPCI:SCC040. Also, the IL1 pathway gene expression was enhanced in unirradiated samples of UPCI:SCC131 and was low and not induced in irradiated UPCI:SCC099 cells. The 9-gene identifier showed a fold change above 1.5 and highly significant q values (**Supplementary Figure 4C**). For association analysis with survival and senescence, the mean over log2 values of all IL1 members was calibrated to the unirradiated control and used as a representative value for IL1 pathway activity. The associations obtained were strong and significant for both radiation qualities (**Figure 4H**).

We also observed that after photon irradiation with 8 Gy, IL1B protein secretion as detected by ELISA nicely coincided with gene expression data (**Figures 4G, I** **)**. In all three cell lines, secretion of IL 1B increased with time after irradiation. In UPCI:SCC131 cells showing the lowest gene expression, also the lowest protein secretion was seen, and protein secretion was detectable in unirradiated samples, which was not the case for the two other cell lines. For Cal33 cells, where gene expression was strong, also protein secretion was strong and UPCI:SCC040 exhibited intermediate levels.

In conclusion, a strong and cell line-specific increase in expression of SASP factors can be detected after irradiation with photons and ^12^C-ions. Especially the IL1 pathway is highly associated with radioresistance and senescence of HPV-negative HNSCC cells.

### IL1A and IL1B Are Not Functionally Involved in Radioresistance and Senescence of HNSCC Cells

Results up to now disclosed a role of IL1 as a strong indicator of radioresistance in HNSCC cells. It was tested whether the strong correlation with survival and senescence also implies that IL1 is functionally involved in these processes. To this end, specific siRNA oligonucleotides were used to perform a single or double knockdown of IL1A and IL1B in the HNSCC cell lines. Efficiency of the knockdown was validated by qRT-PCR and demonstrated a decrease in expression levels from 34% down to 5% (**Supplementary Figure 5A**).

**Figure 5:**
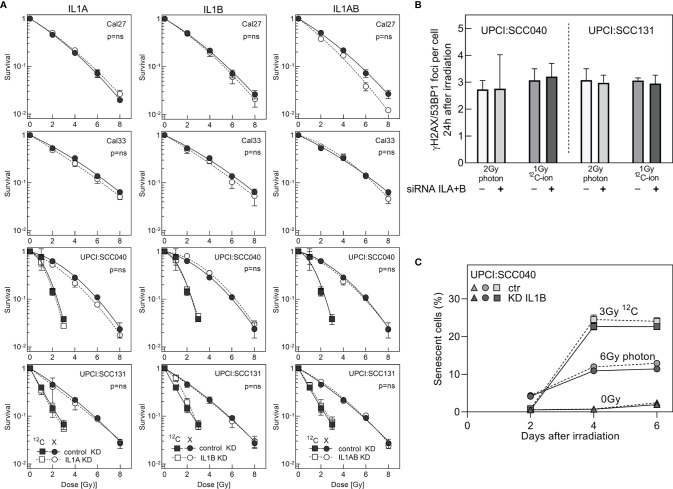
Cell survival after siRNA knockdown of ILA or/and IL1B. **(A)** Colony formation assay was initiated 1 day after transfection. Knockdown and control cells were irradiated with 2–8 Gy photons or 1–3 Gy ^12^C-ions. **(B)** Foci formation in response to 2-Gy photons or 1-Gy ^12^C-ions was examined by γH2AX/53BP1 immunofluorescence 24 h after irradiation. **(C)** Flow cytometry-based analysis of senescence after siRNA knockdown of IL1B in the cell line UPCI:SCC040. IL1B knockdown and control samples were irradiated with 6 Gy photon or 3 Gy ^12^C-ion, and senescence was determined from days 2 to 6 after irradiation. Experiments were performed in triplicates. MV+/- SEM are given. p values were calculated using t-test statistics. p = ns describes no significance was detected.

Surprisingly, neither the IL1A and IL1B single nor double knockdown was found to have an effect on the cellular radioresistance, as determined by colony formation assay (**Figure 5A**). The result was identical for all cell lines examined, and for both radiation qualities. There was also no effect of the knockdown on the number of residual foci as found after irradiation with 2 Gy photon or 1 Gy ^12^C-ion (**Figure 5B**). Likewise, there was no effect of IL1B KD on irradiation-induced senescence (**Figure 5C**), whereby this knockdown was characterized by stable depression of both gene expression and protein secretion over the whole observation period of 5 days (**Supplementary Figure 5B, C** **)**.

Overall, these data demonstrate for the first time that IL1A and IL1B, despite being strong indicators of radioresistance and senescence in HNSCC cells, are not functionally involved in these processes.

## Discussion

Resistance toward irradiation is a frequent cause of therapeutic failure and raises the need to understand the molecular mechanisms of this occurrence. The purpose of this study was to identify determinants of tumor-cell intrinsic radioresistance caused by senescence and the associated SASP in HPV-negative HNSCC cells. We used photons and, for the first time, ^12^C-ion irradiation to examine a panel of five HPV-negative HNSCC cell lines. Clonogenic survival data of both irradiation qualities were correlated with functional and molecular data of DNA damage, senescence, SASP, and IL1 pathway factors. The analysis identified the NFκB-dependent arm of the SASP and the IL1 pathway with its most upstream factors IL1A and IL1B as strong indicators of radioresistance and irradiation-induced senescence. Despite their strong indicative function, both factors were not functionally involved in mechanisms regulating radioresistance.

As expected, ^12^C-ion showed improved cell killing over photons with an RBE of 2–3. The ranking in radiosensitivity is quite similar for both photons and ^12^C-ions, with Cal33 and UPCI:SCC040 being the most radioresistant cell lines, and Cal27, UPCI:SCC099, and UPCI:SCC131 being rather radiosensitive. Concerning DNA damage response, the dynamics of DSB initiation and repair define the increased cytotoxicity of high LET irradiation (32, 42), and this can be observed with our data as well. DSB repair was less efficient after ^12^C-ion than seen after photon irradiation. The increased complexity of DSBs induced by high-LET and subsequent variations in the recruitment of DSB repair pathways were considered to be responsible for this difference (43–45). Residual γH2AX-foci are a well-established marker for assessment of radiosensitivity (46), and our association studies of residual foci with radioresistance showed a moderate correlation. Nevertheless, DSB repair cannot fully explain the underlying nature of variation in radiation response of different cell lines; other factors must contribute to this process.

We could now identify that irradiation-induced senescence is a strong determinant of clonogenic survival not only after photon but also after ^12^C-ion irradiation. The dynamics of senescence were similar for both, but induction was stronger after ^12^C-ion. The alterations in DNA damage response after high LET may be of relevance in this process. The complex clustered damage and the accompanied rearrangement of the chromatin prolong and impair the respective repair of the lesions, and as a biological consequence an increase in senescence may occur (47, 48).

It can be concluded that the cells might undergo senescence irrespective of p53 or p16, since two of the cell lines harbor mutated p53 (Cal27, Cal33), one is absent of p53 (UPCI:SCC040), and only two carry wild-type p53 (UPCI:SCC099, UPCI:SCC131). P16- and p53-independent induction can be observed also in response to other stimuli, such as oncogenes (49), but the underlying mechanisms are not yet clarified. Instead of evaluating the expression of cell-cycle markers, we used a flow cytometry-based assay to assess the lysosomal beta-galactosidase activity, which is an essential hallmark of senescence (50). The detected senescence levels of the five HNSCC cell lines showed a strong correlation with clonogenic survival and demonstrated that radioresistance was high when induction of senescence was strong. Other groups do observe similar results: senescence was identified as a p53-independent mechanism of tumor cells to escape from cytotoxic cell killing after high LET (51). Senescence which was induced after IR of low LET was observed to be a main contributor to tumor progression and tumor cell survival, and the removal of those cells by senolytic drugs could improve the response toward irradiation (9, 52, 53). Our results support the role of senescence as a driver of radiotherapy resistance (54).

Tightly connected to senescence is the expression and secretion of inflammatory factors termed SASP (41). The inflammatory response elicited after IR is composed of a plethora of chemokines and cytokines which are known to contribute to radiation fibrosis in normal tissue and trigger propagation and invasiveness of tumor cells (55). We observed a strong induction of SASP in response to irradiation at the gene expression level. Activation was nearly identical for photon and ^12^C-ions. There was, however, a remarkable cell-line-specific response detectable with a significant separation of irradiated from unirradiated samples according to SASP gene expression. According to literature, the effect detected by us at the RNA level can be observed in various experimental systems, also in tumor specimen, even a long time after irradiation, and is interlinked with radioresistance (56, 57).

The most significant genes identified in our analysis were members of the NFκB-mediated SASP, consisting of factors of the IL1 pathway and CXCR2 chemokine receptor ligands, but also growth factors such as VEGF. Especially the roles of the IL1 pathway are manifold with pivotal contributions to senescence, stress response, and inflammation (20, 21). IL1 is described to suppress immunity and promote tumor growth and metastasis, and it may play a role in carcinogenesis as well (58, 59). This led us to further focus on the factors of this pathway and to study the role in tumor cell-intrinsic radioresistance, which is not yet clarified by current literature. Hierarchical clustering of our RNA-seq data clearly identified a strong association of the IL1 pathway with the radiation response of the HNSCC cell lines. IL1A and IL1B, the activators of the pathway, showed excellent correlations with radioresistance and senescence. This finding suggests that IL1A and IL1B might be involved in these processes.

However, detailed studies revealed that after photon and ^12^C-ion irradiation radioresistance does not depend on IL1A and IL1B. Neither clonogenic survival nor DNA repair were affected by siRNA knockdown of IL1A and IL1B. We found IL1B also being dispensable for initiation of senescence, since knockdown did not have an effect on the occurrence of senescence after irradiation. Lau et al. reported similar findings and confirmed a role for IL1 in SASP expression but not in senescence (60). Apart from our findings, still IL1 pathway members are dominant triggers of inflammation which needs to be considered in tumor treatment. IL1-dependent signaling was shown to elevate oxidative DNA damage and irradiation-induced senescence in inflammatory cancer-associated fibroblasts (iCAFs). Induction of the senescence program included secretion of associated factors, which acted on invasion and metastasis and supplied tumor cells of rectal cancer with a survival advantage after irradiation (61). In turn, the secretion of IL1 members observed in our studies could still be of relevance in a more complex biological setting reflecting also the tumor microenvironment. In HNSCC mouse xenografts, IL1-suppression by the drug anakinra was able to sensitize tumor cells toward EGFR inhibitor treatment (62). In such a context, IL1A could also reach clinical relevance, but clinical trials using anakinra in treatment of HNSCC patients are still outstanding (63).

In a recent study, we already identified NFκB-related components of the SASP as major drivers of radioresistance and inhibition of the SASP by the senomorphic drug metformin was found to sensitize HNSCC tumor cells toward irradiation (9). In particular, the CXCR2-related arm of the SASP was found to be attenuated after treatment. CXCR2 acts as receptor for the chemokines CXCL1–3, 5–8, and its stimulation activates several signaling pathways beneath NFκB, which are involved in tumor cell survival and proliferation (64). Altogether, these results suggest subtle regulatory differences for IL1 and the CXCR2-related SASP. Clearly, more research into this area is needed to define the specific responsible SASP components driving radioresistance in tumor cells.

## Data Availability Statement

The datasets presented in the study are deposited in the GEO repository, accession number GSE198100.

## Author Contributions

Conceptualization, KL, KU, US. Funding acquisition, KL, KU, RE-C, US. Investigation and analysis, DT, FS, JH, KU, RH, SK, US. Supervision, ED, RE-C, US. Manuscript preparation DT, ED, KL, KU, US. All authors contributed to the article and approved the submitted version.

## Funding

The research was supported by grants from the Marburg Research Initiative MIT-Schience (grant number MIT-2017-03), Philipps-University Marburg, and by the Anneliese Pohl Trust, Marburg.

## Conflict of Interest

Author JH and KU are employed by German Research Center for Environmental Health GmbH, Neuherberg, Germany.

The remaining authors declare that the research was conducted in the absence of any commercial or financial relationships that could be construed as a potential conflict of interest.

## Publisher’s Note

All claims expressed in this article are solely those of the authors and do not necessarily represent those of their affiliated organizations, or those of the publisher, the editors and the reviewers. Any product that may be evaluated in this article, or claim that may be made by its manufacturer, is not guaranteed or endorsed by the publisher.
