# Supplementary material for: IL1 Pathway in HPV-Negative HNSCC Cells Is an Indicator of Radioresistance After Photon and Carbon Ion Irradiation Without Functional Involvement
Source: Front Oncol. 2022 Apr 22;12:878675. doi: 10.3389/fonc.2022.878675 (PMC9072779; doi:10.3389/fonc.2022.878675)
Supplement: Supplementary file 1 [file DataSheet_1.pdf]

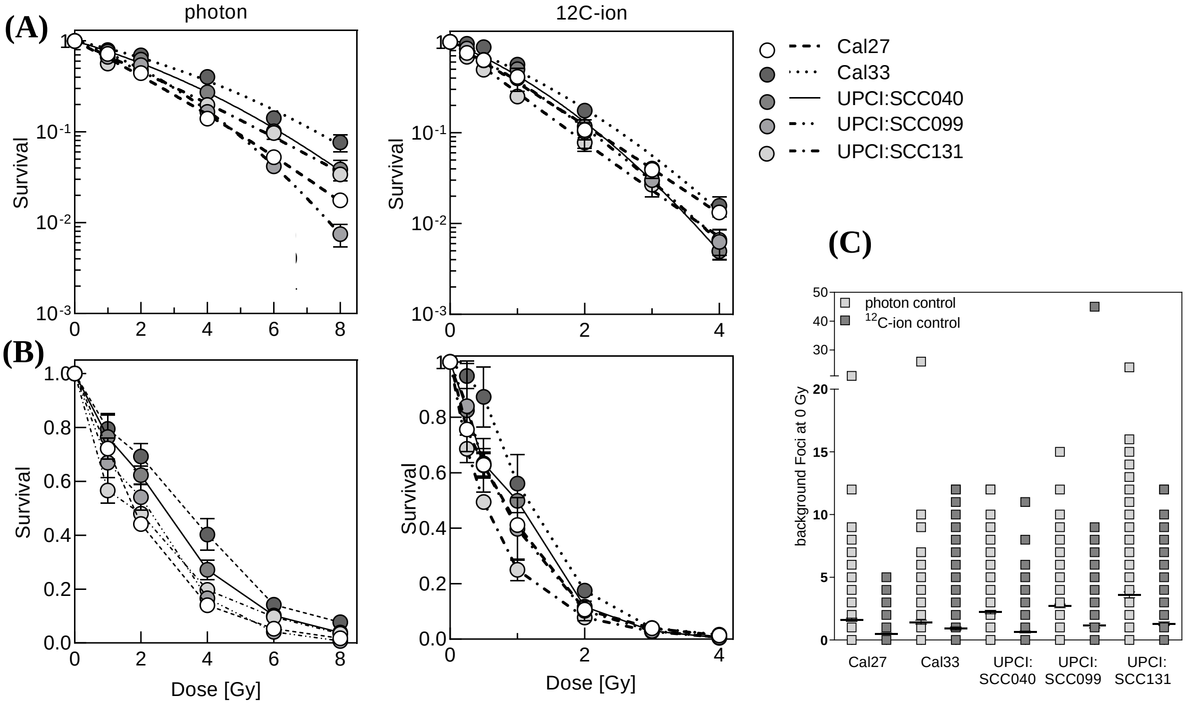

Supplementary Figure 1. Determination of AUC from clonogenic survival data of the HNSCC cell line panel after irradiation with photons or ^{12}C-ions. **(A)** Fitted graph using the linear-quadratic model. **(B)** Graph in linear scale without fitting. MV +/- SEM are indicated. **(C)** Aligned dot plot depicting distribution of background foci in 0 Gy control samples for photon and ^{12}C-ion irradiation.

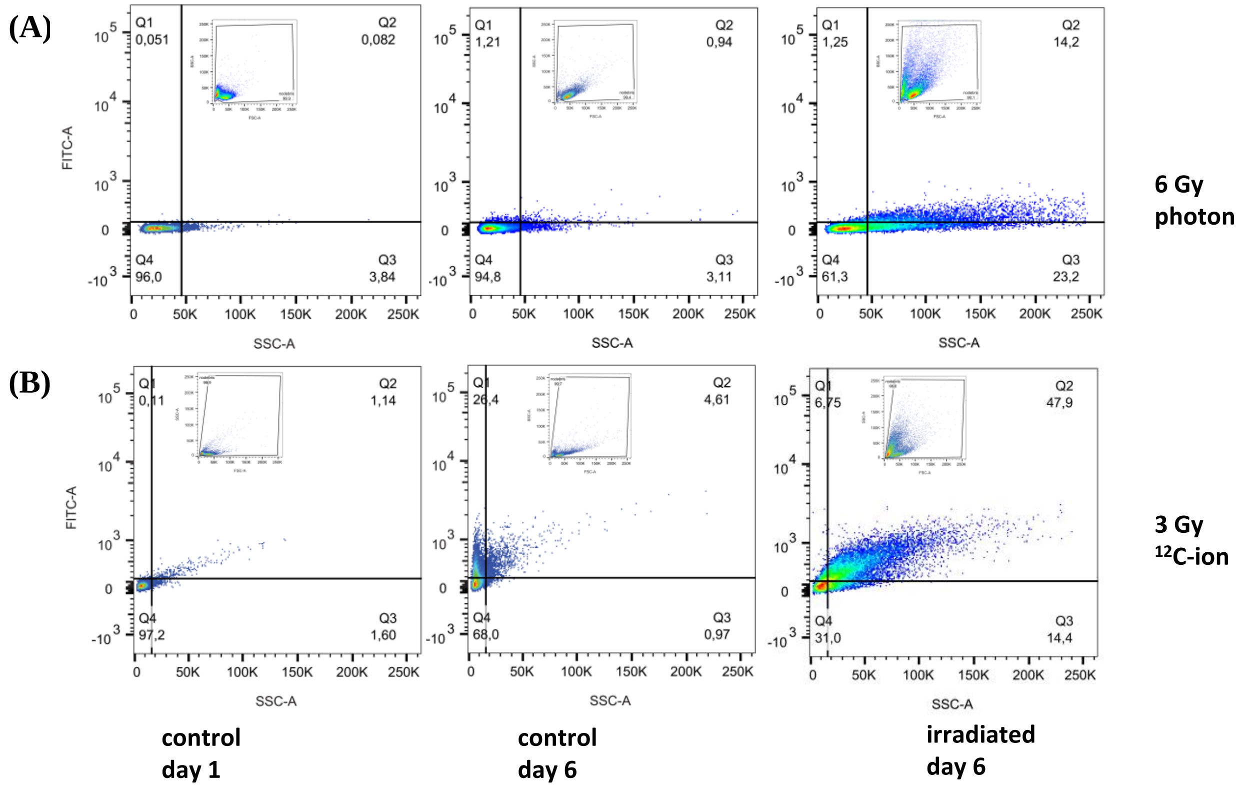

Supplementary Figure 2. Flow cytometric analysis of senescence-associated lysosomal β-galactosidase activity. Gating strategy: After exclusion of cell debris (inlets), gates were set for detection of cells with increased granularity (SSC ^{hi}) and increased β-galactosidase activity (FITC ^{hi}). Gates were set according to the unirradiated control sample of day 1, gating 95%-98% o the cell population in Q4. **(A)** Irradiation with 6 Gy photons and control. **(B)** Irradiation with 3 Gy ^{12}C-ions and control.

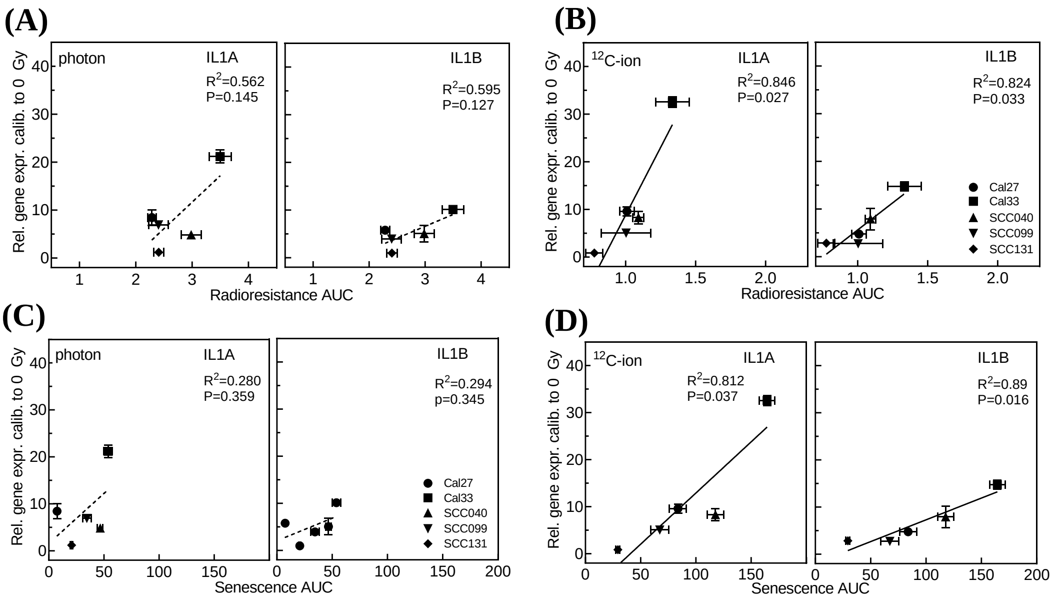

Supplementary Figure 3. Correlation of IL1A and IL1B gene expression with Radioresistance AUC or Senescence AUC. 72h after irradiation with 8 Gy photon or 4 Gy ^{12}C-ion, gene expression was measured by qRTPCR and analysed according to the ddct-method. For normalisation a house keeper matrix (b-actin, Alas and B2M) was used. Each expression value was calibrated to 0 Gy control of the corresponding day. **(A)** Correlation of IL1A and IL1B with Radioresistance AUC after photon and **(B)** after ^{12}C-ion irradiation. **(C)** Correlation of IL1A and IL1B with Senescence AUC after photon and **(D)** after ^{12}C-ion irradiation. T-test statistics were used for p value calculation. P<0.05 are considered significant. R^2: Pearson coefficient. Values are MV+/- SEM. Experiments were performed in three biol. replicates.

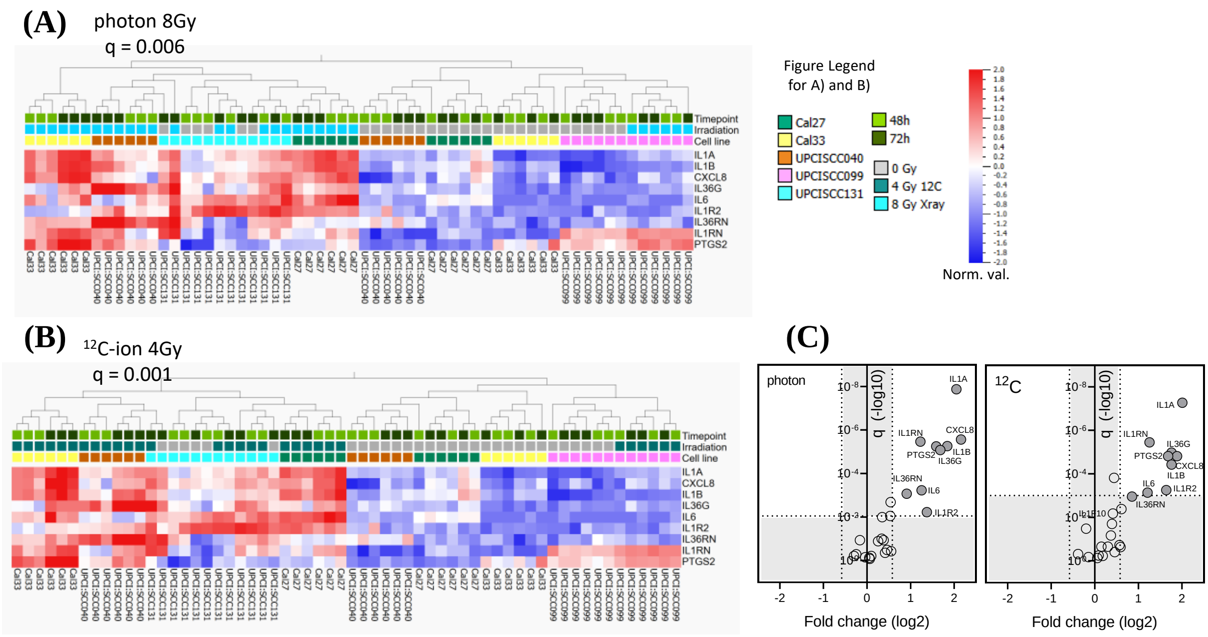

Supplementary Figure 4. IL1 pathway activation after irradiation with photon or ^{12}C-ion. **(A)** Hierarchical clustering (fold change > 1.5) with 9 genes to seperate irradiated from unirradiated samples for photons and **(B)** ^{12}C-ions. **(C)** Plot depicting fold change and q value for significantly differently expressed genes between unirradiated and irradiated samples for photons and ^{12}C-ions. Log2 transformed, normalised (mean=0, var=1) RNA seq data was used. Benjamini Hochberg correction was applied and q<0.05 was considered significant.

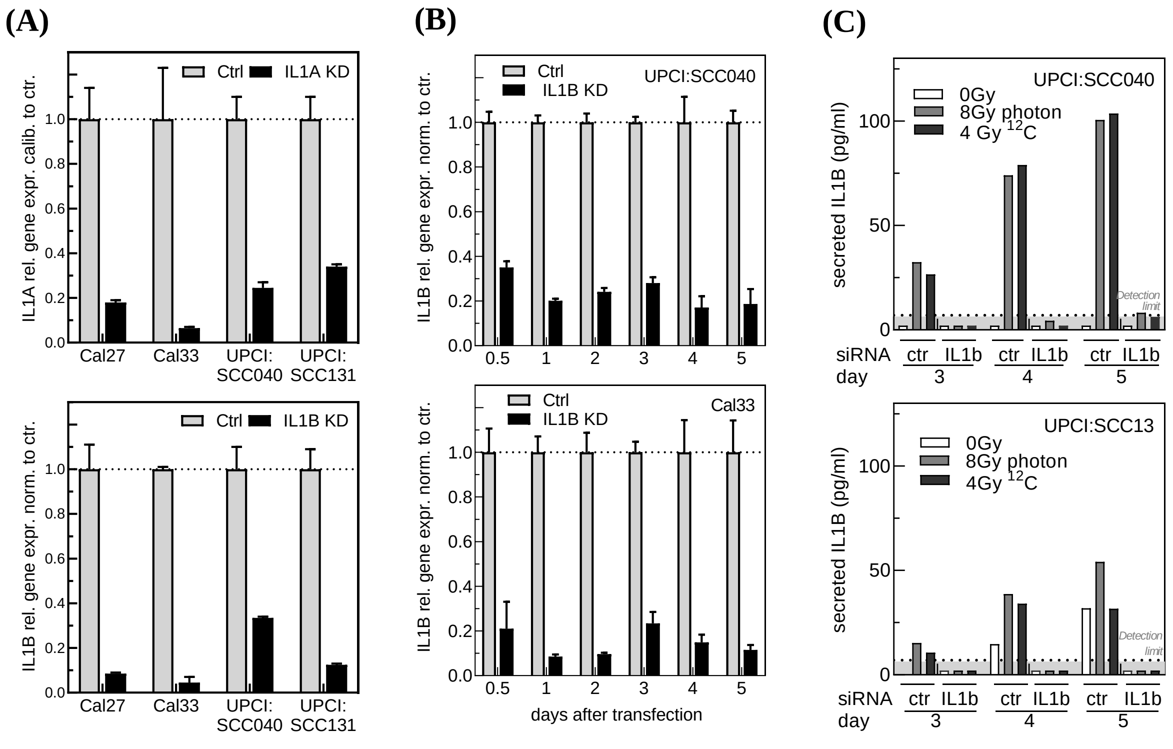

Supplementary Figure 5. Measurement of siRNA transfection efficiency. **(A)** Knockdown validation by qRTPCR. Gene expression of IL1A and IL1B was measured one day after transfection. **(B)** Downregulation of IL1B gene expression by siRNA was measured from day 1 to day 5 after transfection by qRTPCR. **(C)** Inhibition of IL1B protein secretion by siRNA was measured by ELISA. One day after transfection cells were irradiated with 8 Gy photon or 4 Gy ^{12}C-ion, and IL1B secretion was detected from day 3 to day 4 after irradiation. Experiments were performed in triplicates (A) or duplicates (B,C). MV+/-SEM are indicated.
