# Supplementary material for: IL1 Pathway in HPV-Negative HNSCC Cells Is an Indicator of Radioresistance After Photon and Carbon Ion Irradiation Without Functional Involvement
Source: Front Oncol. 2022 Apr 22;12:878675. doi: 10.3389/fonc.2022.878675 (PMC9072779; doi:10.3389/fonc.2022.878675)
Supplement: Supplementary file 2 [file Table_1.docx]

Supplementary Material

# Supplementary Table 1

Sequences of siRNA used for transfection.

| ON-TARGETplus SMARTpool siRNA Mix Target Sequences | | |
| --- | --- | --- |
| *IL1A* | *IL1B* | *Non-Targeting Pool* |
| CCUCACGGCUGCUGCAUUA | CGAUGCACCUGUACGAUCA | UGGUUUACAUGUCGACUAA |
| GAUCAUCUGUCUCUGAAUC | GGUGAUGUCUGGUCCAUAU | UGGUUUACAUGUUUUCUGA |
| CAUGGGUGCUUAUAAGUCA | GAUGAUAAGCCCACUCUAC | UGGUUUACAUGUUGUGUGA |
| GAAAUCAUCAAGCCUAGGU | CAUGGGAUAACGAGGCUUA | UGGUUUACAUGUUUUCCUA |

# Supplementary Table 2

Sequences of primers used for qRTPCR.

| Gene | Primer Sequence Forward 5’-3’ | Primer Sequence Reverse 5’-3’ |
| --- | --- | --- |
| *IL1A* | GCTTCCTGAGCAATGTGAAATACA | CAAATTTCACTGCTTCATCCAGATT |
| *IL1B* | CCTGAHCTGGCCAGTGAAAT | TTTAGGGCCATCAGCTTCAAA |
| *ALAS* | TCCACTGCAGCAGTACACTACCA | ACGGAAGCTGTGTGCCATCT |

# Supplementary Table 3

Values for AUC +/- SD calculated for the HNSCC cell line panel. Radioresistance AUC was derived from the clonogenic survival data set. Senescence AUC was derived from the senescence data set.

|  | **Radioresistance** | | | | **Senescence** | | | |
| --- | --- | --- | --- | --- | --- | --- | --- | --- |
|  | photon | | ^12^C-ion | | photon | | ^12^C-ion | |
|  | AUC | SD | AUC | SD | AUC | SD | AUC | SD |
| Cal27 | 2287 | 0.08 | 1010 | 0.05 | 3.91 | 0.42 | 46.38 | 2.77 |
| Cal33 | 3497 | 0.19 | 1334 | 0.12 | 27.33 | 1.28 | 102.42 | 3.39 |
| UPCI:SCC040 | 2983 | 0.18 | 1092 | 0.04 | 25.94 | 0.96 | 82.64 | 4.65 |
| UPCI:SCC099 | 2403 | 0.17 | 1004 | 0.18 | 17.83 | 1.78 | 47.36 | 4.35 |
| UPCI:SCC131 | 2407 | 0.09 | 0.777 | 0.06 | 13.26 | 0.55 | 22.04 | 1.01 |

# Supplementary Table 4

List of 49 SASP-factor genes extracted from the RNA seq dataset. Genes marked in grey are expressed significantly different between irradiated and unirradiated samples with a fold change > 1.5.

| **SASP genes** | | | | | | |
| --- | --- | --- | --- | --- | --- | --- |
|  | Photon | | | ^12^C | | |
| Gene | p-value | q-value | Fold change | p-value | q-value | Fold change |
| AREG | 9.938E-05 | 5.411E-04 | 2.860E+00 | 1.415E-04 | 8.667E-04 | 2.876E+00 |
| CCL26 | 2.790E-01 | 4.272E-01 | 1.416E+00 | 2.163E-01 | 3.533E-01 | 1.499E+00 |
| CXCL1 | 3.161E-04 | 1.291E-03 | 2.740E+00 | 2.010E-04 | 1.094E-03 | 2.801E+00 |
| CXCL13 | 2.651E-01 | 4.190E-01 | 1.120E+00 | 3.526E-01 | 5.348E-01 | 1.093E+00 |
| CXCL2 | 2.269E-05 | 1.390E-04 | 2.435E+00 | 3.315E-04 | 1.354E-03 | 2.125E+00 |
| CXCL3 | 1.116E-06 | 7.814E-06 | 2.188E+00 | 1.368E-06 | 1.450E-05 | 2.274E+00 |
| CXCL5 | 9.313E-03 | 2.684E-02 | 1.419E+00 | 5.312E-03 | 1.531E-02 | 1.492E+00 |
| CXCL6 | 4.902E-01 | 5.719E-01 | 1.155E+00 | 3.601E-01 | 5.348E-01 | 1.221E+00 |
| CXCL8 | 2.162E-07 | 2.119E-06 | 4.462E+00 | 2.498E-06 | 2.040E-05 | 3.700E+00 |
| CXCR2 | 1.920E-01 | 3.245E-01 | 1.238E+00 | 1.654E-01 | 2.795E-01 | 1.257E+00 |
| CXCR4 | 3.857E-01 | 5.399E-01 | 9.179E-01 | 5.594E-01 | 7.214E-01 | 9.431E-01 |
| CXCR5 | 7.561E-01 | 7.961E-01 | 1.022E+00 | 2.880E-01 | 4.553E-01 | 1.078E+00 |
| EGF | 1.120E-01 | 2.033E-01 | 1.169E+00 | 1.476E-02 | 3.806E-02 | 1.282E+00 |
| EGFR | 4.165E-01 | 5.669E-01 | 1.219E+00 | 3.873E-01 | 5.581E-01 | 1.231E+00 |
| EREG | 1.724E-04 | 7.682E-04 | 3.033E+00 | 1.001E-03 | 3.503E-03 | 2.561E+00 |
| FAS | 1.216E-02 | 3.136E-02 | 1.298E+00 | 1.397E-01 | 2.445E-01 | 1.159E+00 |
| FGF2 | 9.646E-02 | 1.891E-01 | 1.665E+00 | 1.366E-01 | 2.445E-01 | 1.568E+00 |
| FN1 | 8.884E-03 | 2.684E-02 | 2.158E+00 | 7.292E-03 | 1.985E-02 | 2.165E+00 |
| ICAM1 | 1.012E-01 | 1.908E-01 | 1.413E+00 | 1.346E-01 | 2.445E-01 | 1.360E+00 |
| ICAM3 | 4.671E-01 | 5.719E-01 | 9.525E-01 | 6.324E-01 | 7.388E-01 | 1.030E+00 |
| IGFBP2 | 4.351E-01 | 5.719E-01 | 6.775E-01 | 4.051E-01 | 5.604E-01 | 6.531E-01 |
| IGFBP3 | 3.816E-01 | 5.399E-01 | 1.634E+00 | 4.117E-01 | 5.604E-01 | 1.572E+00 |
| IGFBP4 | 6.926E-01 | 7.542E-01 | 1.086E+00 | 8.422E-01 | 8.972E-01 | 1.042E+00 |
| IGFBP5 | 7.636E-01 | 7.961E-01 | 1.056E+00 | 9.595E-01 | 9.705E-01 | 9.913E-01 |
| IGFBP6 | 9.255E-01 | 9.448E-01 | 9.769E-01 | 8.198E-01 | 8.927E-01 | 1.060E+00 |
| IGFBP7 | 4.892E-01 | 5.719E-01 | 1.203E+00 | 7.684E-01 | 8.557E-01 | 1.080E+00 |
| IL15 | 6.373E-01 | 7.097E-01 | 1.050E+00 | 9.197E-01 | 9.589E-01 | 9.905E-01 |
| IL1A | 5.329E-10 | 2.611E-08 | 4.145E+00 | 2.132E-09 | 1.045E-07 | 4.023E+00 |
| IL1B | 8.753E-07 | 7.148E-06 | 3.586E+00 | 8.897E-06 | 6.228E-05 | 3.390E+00 |
| IL6 | 1.653E-04 | 7.682E-04 | 2.381E+00 | 2.601E-04 | 1.159E-03 | 2.313E+00 |
| IL6R | 4.637E-01 | 5.719E-01 | 8.892E-01 | 6.332E-01 | 7.388E-01 | 9.235E-01 |
| IL7 | 9.477E-01 | 9.477E-01 | 9.879E-01 | 6.548E-01 | 7.461E-01 | 1.081E+00 |
| KITLG | 6.430E-02 | 1.313E-01 | 7.451E-01 | 5.816E-02 | 1.187E-01 | 7.392E-01 |
| MMP12 | 1.034E-02 | 2.814E-02 | 6.084E-01 | 3.493E-02 | 7.780E-02 | 6.439E-01 |
| MMP13 | 1.562E-01 | 2.734E-01 | 5.661E-01 | 9.144E-02 | 1.792E-01 | 4.940E-01 |
| MMP14 | 5.435E-01 | 6.193E-01 | 1.119E+00 | 5.503E-01 | 7.214E-01 | 1.115E+00 |
| MMP3 | 1.336E-02 | 3.272E-02 | 8.004E-01 | 6.269E-01 | 7.388E-01 | 9.499E-01 |
| NRG1 | 4.009E-02 | 8.928E-02 | 1.769E+00 | 5.464E-02 | 1.164E-01 | 1.712E+00 |
| PIGF | 4.623E-01 | 5.719E-01 | 1.063E+00 | 9.705E-01 | 9.705E-01 | 1.003E+00 |
| PLAT | 1.733E-02 | 4.045E-02 | 1.477E+00 | 3.088E-03 | 9.457E-03 | 1.637E+00 |
| PLAUR | 4.008E-08 | 6.547E-07 | 4.876E+00 | 1.480E-06 | 1.450E-05 | 4.328E+00 |
| RNASE4 | 3.730E-01 | 5.399E-01 | 9.339E-01 | 5.848E-01 | 7.347E-01 | 1.039E+00 |
| TIMP2 | 3.739E-03 | 1.309E-02 | 1.772E+00 | 2.016E-03 | 6.586E-03 | 1.867E+00 |
| TNFRSF1A | 4.557E-03 | 1.489E-02 | 8.297E-01 | 2.529E-04 | 1.159E-03 | 7.807E-01 |
| TNFRSF1B | 5.906E-02 | 1.258E-01 | 1.439E+00 | 2.984E-02 | 6.963E-02 | 1.523E+00 |
| VEGFA | 1.723E-08 | 4.220E-07 | 1.814E+00 | 6.999E-07 | 1.143E-05 | 1.683E+00 |
| VEGFB | 6.588E-08 | 8.070E-07 | 6.687E-01 | 2.252E-07 | 5.517E-06 | 6.518E-01 |
| VEGFC | 4.234E-04 | 1.596E-03 | 2.780E+00 | 4.629E-04 | 1.745E-03 | 2.779E+00 |
| VEGFD | 2.174E-01 | 3.551E-01 | 8.801E-01 | 2.380E-02 | 5.831E-02 | 7.881E-01 |

# Supplementary Table 5

List of 25 IL1 pathway genes extracted from the RNA seq dataset. Genes marked in grey are expressed significantly different between irradiated and unirradiated samples with a fold change > 1.5.

| **IL1 pathway genes** | | | | | | |
| --- | --- | --- | --- | --- | --- | --- |
|  | Photon | | | ^12^C | | |
| Gene | p-value | q-value | Fold change | p-value | q-value | Fold change |
| CASP1 | 0.51675445 | 0.61518387 | 0.81200994 | 0.40714052 | 0.48469109 | 0.76626327 |
| CCL2 | 0.26638476 | 0.36997884 | 1.46655602 | 0.31578831 | 0.39473538 | 1.37277885 |
| CXCL8 | 2.16E-07 | 2.70E-06 | 4.46230754 | 2.50E-06 | 1.53E-05 | 3.6997852 |
| IL18BP | 0.71952046 | 0.74950048 | 1.03555705 | 0.49322628 | 0.56048441 | 1.06303332 |
| IL18R1 | 0.05127759 | 0.09861075 | 1.26155646 | 0.0404908 | 0.06748467 | 1.29052037 |
| IL1A | 5.33E-10 | 1.33E-08 | 4.14465942 | 2.13E-09 | 5.33E-08 | 4.02277916 |
| IL1B | 8.75E-07 | 5.47E-06 | 3.58581725 | 8.90E-06 | 3.71E-05 | 3.39015666 |
| IL1F10 | 0.08211007 | 0.12829699 | 1.19204358 | 0.00317271 | 0.00660981 | 1.3337565 |
| IL1R1 | 0.82941744 | 0.82941744 | 1.04551087 | 0.54009006 | 0.58705441 | 1.13428114 |
| IL1R2 | 0.00238112 | 0.00595281 | 2.59108249 | 0.00017819 | 0.00055685 | 3.11581345 |
| IL1RAP | 0.54234404 | 0.61630005 | 1.06971171 | 0.73201173 | 0.73201173 | 1.04053525 |
| IL1RAPL1 | 0.0039127 | 0.0088925 | 1.44693739 | 0.00182368 | 0.00414474 | 1.52672491 |
| IL1RAPL2 | 0.33353212 | 0.43885806 | 1.332991 | 0.17522777 | 0.23056286 | 1.50374356 |
| IL1RL2 | 0.22042308 | 0.32415159 | 1.37495515 | 0.12019566 | 0.18780571 | 1.47876103 |
| IL1RN | 4.06E-07 | 3.38E-06 | 2.33576664 | 2.83E-07 | 3.54E-06 | 2.38790357 |
| IL33 | 0.40742166 | 0.50927708 | 0.84254055 | 0.63894134 | 0.66556389 | 0.90425853 |
| IL36B | 0.00480856 | 0.01001782 | 1.27364746 | 4.28E-05 | 0.00015291 | 1.35699694 |
| IL36G | 1.97E-06 | 8.22E-06 | 3.20490457 | 1.24E-06 | 1.04E-05 | 3.38728601 |
| IL36RN | 0.00027141 | 0.00084817 | 1.87930834 | 0.00043796 | 0.0010949 | 1.80605951 |
| IL37 | 0.65036227 | 0.70691551 | 0.97064483 | 0.15968659 | 0.22178693 | 1.11073347 |
| IL6 | 0.00016531 | 0.00059038 | 2.38086186 | 0.00026011 | 0.00072253 | 2.31253149 |
| IRAK4 | 0.06918756 | 0.1153126 | 0.89290333 | 0.01845904 | 0.03296257 | 0.86987798 |
| MYD88 | 0.00074317 | 0.00206436 | 1.45671927 | 0.01037739 | 0.01995652 | 1.31313564 |
| NFKBIA | 0.06850853 | 0.1153126 | 1.30280779 | 0.15577239 | 0.22178693 | 1.23032049 |
| PTGS2 | 1.13E-06 | 5.63E-06 | 3.00504403 | 3.06E-06 | 1.53E-05 | 3.20780725 |
